# Thulium laser versus standard transurethral resection of the prostate: A randomized prospective trial

**Published:** 2008

**Authors:** Deepak Dubey, K. Muruganandham

**Affiliations:** Department of Urology, SGPGI, Lucknow, India

## SUMMARY

In this prospective trial the authors randomized 100 consecutive patients to receive either a TURP (n = 48) or Thulium Laser Prostatectomy (n = 52). All patients were preoperatively assessed with subjective symptoms score, International Index of Erectile Function questionnaire, and complete urodynamic evaluation. Preoperative and perioperative parameters at 1-, 6-, and 12-months follow-up were also evaluated. All complications were recorded. TmLRP-TT was significantly superior to TURP in terms of catheterization time (45.7 ± 25.8 h vs. 87.4 ± 33.8 h, *P* < 0.0001), hospital stay (115.1 ± 25.5 h vs. 161.1 ± 33.8 h, *P* < 0.0001), and drop in hemoglobin (0.92 ± 0.82 g/dl vs. 1.46 ± 0.65 g/dl, *P* < 0.001), whereas it required equivalent time to perform (46.3 ± 16.2 vs. 50.4 ± 20.7 min, *P* > 0.05). TmLRP-TT and TURP resulted in a significant improvement from baseline in terms of subjective symptoms scoring and urodynamic finding, but no significant difference was found between the two groups. Late complications were also comparable. TmLRP-TT is an almost bloodless procedure with high efficacy and little perioperative morbidity. TmLRP-TT is superior to TURP in safety and is as efficacious as TURP in one-year follow-up. It is a promising technology in the clinical practice field.

## COMMENTS

### Is TURP out?

The advent of modern laser technology continues to offer a serious threat to the current gold standards for treating BPH, viz. TURP/Open prostatectomy. In a randomized trial comparing TURP with HoLEP, Tan *et al*.[1] demonstrated that HoLEP is superior to TURP in improving urodynamic bladder obstruction along with shorter catheterization time and decreased blood loss. In a recent randomized trial,[2] HoLEP showed better outcomes as compared to open prostatectomy for adenomas larger than 100 g over a long-term follow-up of five years. However, the learning curve for HoLEP is steep, which has prevented many urologists from accepting this technique. KTP green light laser has also demonstrated equivalence to TURP in a prospective clinical trial,[3] albeit with a follow-up of only six months. Similarly KTP laser prostatectomy has been found to be equally effective and safe as compared to open transvesical prostatectomy in a randomized prospective trial.[4]

In this statistically well-powered randomized prospective trial the authors have compared Thulium laser prostatectomy (ThLRP) to TURP. Over a follow-up of 12 months, they reported equal efficacy of the two techniques and decreased catheterization time, blood loss and hospital stay in favor of the Thulium laser. Unlike KTP, ThLRP retrieves prostatic tissue for biopsy, has a shorter learning curve than HoLEP. The operative technique most closely resembles TURP as compared to other lasers. Before this technique becomes widely accepted, we await experience from other centers with longer follow-up. However, it is becoming increasingly evident that TURP is facing genuine competition and might be replaced as the gold standard in minimally invasive surgery of the prostate.

## References

[1] Tan AH, Gilling PJ, Kennett KM, Frampton C, Westenberg AM, Fraundorfer MR (2003). A randomized trial comparing holmium laser enucleation of the prostate with transurethral resection of the prostate for the treatment of bladder outlet obstruction secondary to benign prostatic hyperplasia in large glands (40 to 200 grams). J Urol.

[2] Kuntz RM, Lehrich K, Ahyai SA (2008). Holmium laser enucleation of the prostate versus open prostatectomy for prostates greater than 100 grams: 5-year follow-up results of a randomised clinical trial. Eur Urol.

[3] Bachmann A, Schürch L, Ruszat R, Wyler SF, Seifert HH, Müller A (2005). Photoselective vaporization (PVP) versus transurethral resection of the prostate (TURP): A prospective bi-centre study of perioperative morbidity and early functional outcome. Eur Urol.

[4] Alivizatos G, Skolarikos A, Chalikopoulos D, Papachristou C, Sopilidis O, Dellis A (2009). Transurethral photoselective vaporization versus transvesical open enucleation for prostatic adenomas >80 ml: 12-mo results of a randomized prospective study. Eur Urol.

